# Widespread detection of Polyethylene glycol reveals chronic human exposure through pharmaceutical drugs

**DOI:** 10.64898/2026.09.11.750999

**Published:** 2026-09-18

**Authors:** Harsha Gouda, Patricia Kelly, Luke Whiley, Daniel Gómez-Pérez, Anna Mascellani Bergo, Wilhan Donizete Gonçalves Nunes, Elena Chekmeneva, Ada H.Y. Yuen, Mark David, Shona McKirdy, Andrew Nelson, Darren L. Smith, Haoqi Nina Zhao, Jeong In Seo, Edward J. Mathias, Gillian Farrell, Toon Scheurink, Michael Strobel, Helena Mannochio-Russo, Konstantinos Gkikas, Mingxun Wang, Jaroslav Havlik, Christopher Quince, Zoltan Takats, Matthew Lewis, Konstantinos Gerasimidis, Nicholas J. W. Rattray, Pieter C. Dorrestein

## Abstract

Polyethylene glycol (PEG) is a synthetic polymer ubiquitous in pharmaceuticals, personal care products, food additives, and industrial manufacturing. Despite its widespread use and potential importance as an exposure chemical, the prevalence of PEG exposure and its excretion in human populations remain largely uncharacterized. Moreover, PEG detected in human biofluids is frequently assumed to arise from analytical contamination during sample preparation, potentially obscuring its contribution to the human exposome and confounding metabolic phenotyping studies. Here, we show that PEG as a component of the human xenobiotic exposome and is further metabolized into PEG hydoxy acid and diacid metabolites in humans. We further find that PEG exposure is associated with alterations in microbiome composition and short-chain fatty acid metabolism, suggesting its biological impact of exposure. We identify PEG exposure in approximately 2.3% of publicly available metabolomics data files and provide a reusable 85,484 candidate PEG and PEGylated MS/MS spectral library for future use for the metabolomic community. Together, these findings establish PEG in human biofluids can reflect genuine exposure, and that PEG exposure is neither metabolically inert nor biologically silent.

## Full Text Availability

The license terms selected by the author(s) for this preprint version do not permit archiving in PMC. The full text is available from the preprint server.

